# Effect of L-PRF and autogenous bone grafts for immediate implant placement in post tooth extract socket

**DOI:** 10.6026/973206300200639

**Published:** 2024-06-30

**Authors:** Bhavna Jha Kukreja, Abdulrahman Ahmed Aseri, Arun Vashisht, Hemal Patel, Parul Singhal, Naina Pattnaik, Mahesh Ghadage, Dipooja Patil

**Affiliations:** 1 Preventive Dental sciences Department, College of Dentistry, Gulf Medical University, Ajman, UAE; 2 Periodontics, Department of Preventive Dental Sciences, Faculty of Dentistry, Najran University, Saudi Arabia; 3 BDS, MDS, PG Prosthodontics, Gimmesmile PLLC, 2935 Tory hill lane, Sugarland Texas 77478, North America; 4 Department of Public Health Dentistry, Faculty of Dental Science, Dharmsinh Desai University, Nadiad, Gujarat, India; 5 Departmentof Prosthodontics, RUHS College of Dental Sciences, Jaipur, India; 6 Department of Periodontics and Oral Implantology, Kalinga Institute of Dental Science, KIIT Deemed to be University, Patia, Bhubaneswar, Odisha, India; 7 Department of Prosthodontics and Crown and Bridge, Bharati Vidyapeeth (Deemed to be University) Dental College and Hospital, Navi Mumbai, Maharashtra, India; 8 Department of Conservative Dentistry and Endodontics, Bharati Vidyapeeth (Deemed to be University) Dental College and Hospital, Navi Mumbai, India

**Keywords:** Immediate dental implant, L-PRF, autogenous bone grafts

## Abstract

The clinical outcomes of bone augmentation substances in immediate dental implant (IDI) placement are of interest to dentists. Hence, we evaluated and compared the effectiveness of L-PRF and autogenous bone grafts in immediate implant placement in tooth extract socket. Hence, assessment of periimplantis pocket depth, assessment of tissue biotype, implant stability and marginal bone loss at one month, three months, and six months follow up was completed. The tissue biotype values at one month, 3 month and 6 month follow up revealed that tissue biotype increased in each category as the time increased in all categories. We found that all three techniques were found to have good clinical outcomes regarding immediate implant placement in fresh tooth extraction socket, however the outcomes were better in the case L-PRF.

## Background:

Given that, they significantly reduce treatment times, immediate dental implants (IDI) have changed the practice of implant dentistry. Schulte and Heimke first documented the immediate implantation of dental implants in a tooth extraction socket (TES) in 1976 [1, 2-3]. Due to its many advantages, including improved soft tissue visual appeal and faster treatment durations because it requires fewer surgical procedures, the technique is currently widely recognized [4, 5- 6]. Because of variations in the shape, dimensions, and morphology of TES, an IDI implanted in a newly extracted alveolus may cause a separation between the surface of the implant and the wall of extraction socket [5- 7]. Subsequent remodeling causes resorption and occasionally exposes part of the implant, which has a negative aesthetic effect [8, 9-10]. Smaller gaps of less than 2mm heal on their own, while bigger gaps may need to be filled with barrier films and/or bone grafts, or both, to promote improved healing [11, 12-13]. With varied degrees of effectiveness and downsides, platelet-rich plasma (PRP), bone replacements, auto grafts have been tested together with changed surgical approaches to boost the success of IDI [14, 15, 16-17]. Leukocyte-platelet-rich fibrin (L-PRF) is another possible regenerative tactic for encouraging and augmenting the repair mechanism in the post extraction alveolus. It is already widely used in oral surgery, particularly for the preservation of the alveolar ridge [15-18]. Due to the gradual dispersion of fibrin matrix growth stimulants, L-PRF, operates well in terms of promoting healing since it comprises the majority of growth factors, leukocytes and platelet agglomerates [19, 20- 21]. Because PRF may form membranes, it can function as a bio-barrier and preserve sockets sufficiently. This autologous regenerative biomaterial can be prepared quickly and at a low cost [22, 23, 24-25]. Paradoxically, there is now very little data to support the clinical application of L-PRF as a biomaterial for rapid implant insertion. Therefore, it is of interest to evaluate and compare the effectiveness of L-PRF and autogenous bone grafts in immediate implant placement in tooth extract socket.

## Materials and Methods:

The purpose of this study was to assess the effectiveness of L-PRF and autogenous bone grafts in the immediate TES in locations selected for IDI, both clinically as well as radiographic ally.

## The requirements for inclusion were:

[1] Persons with good oral hygiene who are between the ages of 18 and 65

[2] Enough volume in the bones

[3] Thick trabecular bone and porous cortical bone at the location of the new implant

[4] Absence of an acute infection

## The following conditions were listed as urgent implant candidates:

[1] Root fractures,

[2] Severely deteriorated roots or root resorptions.

## The following were the exclusion standards for study participants:

[1] Patients who don't practice good dental hygiene

[2] Smokers as of now

[3] Individuals with a systemic sickness or ailment that might impede with implant placement.

[4] Individuals with periapical diseases in their teeth and traumatically closed mouths

A total of 54 immediate implant sites were included.

Category 1: IDI without L-PRF membrane and autogenous bone graft (n=18)

Category 2: IDI along with the application of L-PRF membrane (n=18)

Category 3: IDI with autogenous bone grafts (n=18)

The same technician implanted each implant, which had a diameter that varied between 3.75 to 4.5 mm. The existence of native bone, bone design, quantity, quality, length, and height, as well as the adequate distance accessible coronal to nasal floor and the maxillary antrum floor were taken into consideration when analyzing study casts and pre-treatment CBCT radiographs. Phase I therapy was administered to the patients, consisting of root planing, scaling, and polishing. This was carried out before the implants were placed, and advice on good dental hygiene was provided. Every patient practiced careful plaque management, maintaining a plaque index rating of less than one. Three categories were randomly selected from the implant sites.

## L-PRF preparation:

The day before the IDI surgery, the patient's venous blood was used to prepare the L-PRF. The patient gave up about 10 millilitres of blood, which was obtained in sterile Vacuette tubes. Blood was collected and centrifuged on a manual mode for 12 minutes at a speed of 2700 rpm. In order to construct the L-PRF membrane, the L-PRF clot was moved to the PRF box's pressboard, and after a minute, the compressor lid was left in place. This produced an L-PRF membrane with a consistent thickness. By taking this step, the membrane is guaranteed to stay hydrated for a few hours.

## Surgical procedure:

In order to provide local anesthetic intra orally, the necessary nerve blocks were carried out in accordance with the surgical site's anatomic requirements. A 2% lignocaine hydrochloride solution mixed with 1: 80,000 adrenalines was injected. To save the alveolar bone, the teeth were extracted carefully using a piezotome or forceps. The structural integrity of the lingual and buccal cortical plates was carefully maintained. To make sure that no foreign material or bone chip remains, the tooth extraction socket was irrigated with sterile saline and properly curetted. .Drilling was carried out at 600-800 rpm in the predefined direction, with the assistance of an implant's surgical drill guide. Using a physio-dispenser (ACTEON), the osteotomy site was prepared with extensive saline irrigation. Depending on the chosen implant size, successive drilling was done until the required dimensions were reached. Implant insertion was done by hand using a rachet and manual key. To obtain satisfactory primary stability, dental implants were positioned in the post tooth extraction socket 2-3 mm beyond the apex. [13] Every implant's length and width was determined based on preoperative radiography and clinical criteria. All implants were positioned either slightly beneath or at the height of the crestal bone. When the desired level of resistance for sitting was reached; each implant was manually positioned and rotated clockwise. Implant seating was completed, resulting in the coronal portion of the collar resting at or below the alveolar ridge's crestal bone level. The implant body was then covered with a screw, and the implantation procedure was carried out.

In category 2 patients, the L-PRF membrane was inserted while in category 3 autogenous bone graft were applied in order to close the space between the surface of implant and wall of extraction socket. The edges of the flaps were moved, and tension-free sutures were used, using 3-0/4-0 braided silk sutures and simple interrupted sutures.

At one month, three months, and six months, different clinical and radiographic characteristics were collected, and patients were brought back on the tenth day for the removal of sutures. After three months, a second step of surgery was performed. Where tissue adaptation was insufficient, gingival flaps were implanted, and prostheses were subsequently provided. There was assessment of periimplantis pocket depth, assessment of tissue biotype, implant stability and marginal bone loss.

## Statistical analysis:

After entering the data into the Microsoft Excel 2000 program, descriptive statistics were carried out by figuring out the continuous variables' mean and standard deviation. The unpaired t-test and the Chi-square test were the statistical methods employed. P < 0.05 was regarded as statistically significant for all statistical analyses carried out with the Statistical Package for the Social sciences (SPSS) version 22.0.

## Results:

The peri-implant probing depth at 3 months follow up in category 1, category 2 and category 3 was 1.71±0.56, 1.54±0.61 and 1.57±0.61 respectively. Similarly, at 6 month follow up, the values were 2.16±0.61, 1.92±0.65 and 1.95±0.65 respectively. Peri-implant probing depth was high in immediate implants with no bone augmentation procedure while it was low in immediate implants with L-PRF. The peri-implant probing depth in immediate implants with autogenous bone grafts was in between the above two categories (Table 1).

The tissue biotype at baseline was comparable in all three categories. The tissue biotype values at one month, 3 month and 6 month follow up revealed that tissue biotype increased in each category as the time increased in all categories. It was also observed that values of tissue biotype were greatest in category of immediate implants with L-PRF while it was lowest in immediate implants with no bone augmentation substitutes. The values for immediate implants with autologous bone grafts were between the two categories discussed above (Table 2).

The values of implant stability were comparable between the category 1,2 and 3 at baseline. There was significant increase in implant stability at 3 months and 6 months follow up in each category. The implant stability at 3 month and 6 month follow up in category 1 was 0.33 ± 0.25 and 0.44 ± 0.61. While in case of category 2, the values were 0.55±0.81 and 0.33±0.50 respectively at 3 month and 6 month follow up. Finally the values were 0.44±0.65 and 0.33±0.50 at 3 month and 6 month follow up in category 3. It was also observed that values of tissue biotype were greatest in category of immediate implants with L-PRF while it was lowest in immediate implants with no bone augmentation substitutes. The values for immediate implants with autologous bone grafts were between the two categories discussed above (Table 3). The marginal bone loss was almost similar in all three categories at the time of placement of immediate implants. The marginal bone loss at 3 month and 6 month follow up was reported to be high in category of no autologous bone grafts followed by category of autologous grafts and L-PRF. (Table 4).

## Discussion:

The technique of immediate implant placement in post extraction socket is currently well-known due to its various benefits, which include quicker treatment durations due to fewer surgical procedures needed and increased soft tissue visual attractiveness [11-15]. A separation between the implant's surface and the extraction socket wall may result from an IDI implanted in a recently extracted alveolus due to differences in the size, shape, and morphology of TES [12-16]. Resorption brought on by further remodeling occasionally exposes a portion of the implant, which is unsightly. Since L-PRF contains most growth factors, leukocytes, and platelet agglomerates, it functions well in terms of promoting healing because of the gradual dispersion of fibrin matrix growth stimulants [16-18]. PRF has the ability to create membranes, which allows it to act as a bio barrier and adequately maintain sockets. This autologous regenerative biomaterial is inexpensive and easily manufactured [19-22]. Ironically, there is currently very little evidence to support the use of L-PRF as a biomaterial for quick implant insertion in clinical settings [13-18]. This study was therefore carried out to evaluate and compare the effectiveness of L-PRF and autogenous bone grafts in immediate implant placement in tooth extract socket. We found that all three techniques were found to have good clinical outcomes regarding healing process; however the outcomes were better in case of IDI with L-PRF. The marginal bone loss and peri-implant pocket depth at 3 month and 6 month follow up was reported to be high in category of no bone grafts followed by category of autogenous bone grafts and L-PRF. In our study the tissue biotype at baseline was comparable in all three categories. The tissue biotype values at one month, 3 month and 6 month follow up revealed that tissue biotype increased in each category as the time increased in all categories. It was also observed that values of tissue biotype were greatest in category of immediate implants with L-PRF while it was lowest in immediate implants with no bone augmentation substitutes. The values for immediate implants with autologous bone grafts were between the two categories discussed above. The findings of our study are having similarity with findings of some studies that showed improved clinical outcomes on applying L-PRF membrane during immediate dental implant placement in post extraction socket [12-19]. Platelet-rich plasma (PRP), bone replacements, auto-grafts, and have been tested in conjunction with modified surgical techniques to increase the success of IDI, with varying degrees of efficacy and drawbacks [14-23]. Another potential regenerative strategy for promoting and enhancing the healing mechanism in the post extraction alveolus is leukocyte-platelet-rich fibrin (L-PRF). In oral surgery, it is already frequently utilized, especially to preserve the alveolar ridge [22-24]. Since L-PRF contains most growth factors, leukocytes, and platelet agglomerates, it functions effectively in encouraging healing because of the fibrin matrix growth stimulants' slow dispersion. [11-19]. PRF has the ability to create membranes, which allows it to act as a bio barrier and adequately maintain sockets. This autologous regenerative biomaterial is inexpensive and easily manufactured [4-8]. The findings of our study are not in accordance with some other studies because these studies have shown no difference in clinical outcomes on using L-PRF and autogenous bone graft [25-26]. The reason of difference may be small sample size of these studies.

In our study, the values of implant stability were comparable between the category 1, 2 and 3 at baseline. There was significant increase in implant stability at 3 months and 6 months follow up in each category. The implant stability at 3 month and 6 month follow up in category 1 was 0.33±0.25 and 0.44±0.61. While in case of category 2, the values were 0.55±0.81 and 0.33±0.50 respectively at 3 month and 6 month follow up. Finally the values were 0.44±0.65 and 0.33±0.50 at 3 month and 6 month follow up in category 3. It was also observed that values of tissue biotype were greatest in category of immediate implants with L-PRF while it was lowest in immediate implants with no bone augmentation substitutes. The values for immediate implants with autogenous bone grafts were between the two categories discussed above.

The findings are supported by some studies while some studies don’t support findings of our study [14, 16, 17, 25, 26]. IDI have revolutionized implant dentistry since they drastically shorten treatment times. The technique is currently well-known due to its various benefits, which include quicker treatment durations due to fewer surgical procedures needed and increased soft tissue visual attractiveness [13-16]. A separation between the implant's surface and the extraction socket wall may result from an IDI implanted in a recently extracted alveolus due to differences in the size, shape, and morphology of TES. Resorption brought on by further remodeling occasionally exposes a portion of the implant, which is unsightly [21-26].

## Conclusion:

Both L-PRF and autogenous bone grafts produced better healing and clinical outcomes in immediate implant placement in post tooth extract socket, however L-PRF gave better clinical outcomes when compared to autogenous bone grafts.

## Figures and Tables

**Table 1:** Comparison of peri-implant probing depth at different time points among three groups

	**Peri-implant probing depth**	
	**3 months**	**6 months**
Category 1	1.71±0.56	2.16±0.61
Category 2	1.54±0.61	1.92±0.65
Category 3	1.57±0.61	1.95±0.65
P value	0.426	

**Table 2:** Comparison of tissue biotype at different time points among three categories

	**Tissue biotype**			
	**Baseline**	**1 month**	**3 months**	**6 months**
Category 1	1.67±0.84	1.44 ±0.61	1.55±0.84	1.67±0.84
Category 2	1.68±0.9	1.78 ±0.98	1.91 ±0.14	2.11 ±0.98
Category 3	1.63±0.9	1.57±0.96	1.69±0.17	1.81±0.98
P value	0.234	0.431	0.567	0.142

**Table 3:** Comparison of implant stability at different time points among three categories

	**Baseline**	**3 months**	**6 months**
Category 1	0.12 ±0.44	0.33±0.25	0.44±0.61
Category 2	0.14±0.51	0.55±0.81	0.33±0.50
Category 3	0.13±0.64	0.44±0.65	0.33±0.50
P value	0.761	0.365	0.621

**Table 4:** Comparison of marginal bone loss at different time points between among three categories

	**Baseline**	**1 month**	**3 months**
Category 1	0.26±0.31	0.57±0.42	0.92 ±0.57
Category 2	0.27±0.33	0.53±0.67	0.64±0.49
Category 3	0.25±0.41	0.49±0.42	0.78±0.61
P value	0.435	0.231	0.678
